# Simulation of optical radiation force distribution in interference patterns and necessary conditions for chiral structure formation on dielectrics

**DOI:** 10.1038/s41598-022-18615-9

**Published:** 2022-09-10

**Authors:** Yoshiki Nakata, Koji Tsubakimoto, Hiroyuki Shiraga, Noriaki Miyanaga, Yuki Kosaka, Masataka Yoshida

**Affiliations:** 1 Institute of Laser Engineering, Osaka University, 2-6 Yamadaoka, Suita, Osaka 565-0871 Japan; 2 Institute for Laser Technology, 1-8-4 Utsubo-honmachi, Nishi-ku, Osaka, 550-0004 Japan; 3 JGC Holdings, 2-3-1 Minato Mirai, Nishi-ku, Yokohama, Kanagawa 220-6001 Japan; 4 Osaka Gas Co., Ltd., 4-1-2 Hiranomachi, Chuo-ku, Osaka, 541-0046 Japan

**Keywords:** Laser material processing, Polymers, Surface patterning

## Abstract

A chiral structure is formed by the optical radiation force induced by a circularly polarized light that has spin angular momentum; chiral structures are expected to be used for light control devices and molecular chirality discrimination devices. In this paper, we clarify the relationship between the differences in the distributions of the optical radiation force and the possibility of formation of chiral structures. We first simulate the optical radiation force distribution in the case of a Gaussian beam that successfully forms a chiral structure. Given a vector ${\varvec{r}}$ with a centre of the light spot $\mathrm{O}$ and polar coordinates $R(\left|{\varvec{r}}\right|, \theta )$, and an optical radiation force vector ${\varvec{F}}$ at $R$, the angle ${\theta }^{\mathrm{^{\prime}}}=\mathrm{\angle }({\varvec{r}}, {\varvec{F}})$ and $\left|{\varvec{F}}\right|$ must be constant with respect to the declination angle $\theta$ for a chiral structure to form. These conditions are fulfilled in the case of a 6-beam interference pattern, but not in the case of a 4-beam interference pattern, which is consistent with the result that no chiral structure is formed in the latter case. The equations derived for simulation of optical radiation force distribution can be used for any optical intensity distribution, and will be of great help in the research of any dielectrics deformation.

## Introduction

The optical radiation force can deform dielectric material surfaces, such as photo-isometric azopolymers, due to their electric susceptibility, and the shape can be controlled by the artificial distribution of light^1–4^. Recently, chiral structures have been fabricated according to the spiral gathering force distribution induced by a focused circularly polarized beam^5–7^. In these experiments, spiralling on an azopolymer occurred due to the optical radiation force induced by a circularly polarized Gaussian beam. On the other hand, interference pattern processing methods can form nanometre- or micron-sized structures such as nanowhiskers^8,9^, nanodrops^10,11^, grating^12,13^, and grating inside an active medium^14,15^, which have been fabricated with a single laser beam exposure. By combining these techniques, a chiral structure in an array was successfully fabricated by using an interference pattern of circularly polarized 6-beams facing each other symmetrically^16^.

Chiral structures are expected to be used as light control devices^17^ and molecular chirality discrimination devices^18^. In these applications, the arraying of the structures increases the functionality of individual chiral structures, enhances signals that are difficult to detect, and, furthermore, allows us to experiment with the control of light waves through a coherent structure. The array structure can be formed by repeating a single process to form a chiral structure with a Gaussian beam, but the uniformity deteriorates with the shot-to-shot fluctuation of the laser, and the control of the processing position must be accurate at the wavelength level for a coherent structure. Therefore, it is advisable to use the characteristics of interference patterns with precise periodicity in the formation process. In this case, the interference pattern should be limited to a periodic array of spot shapes, which are formed for 3, 4 and 6 beams^19^. Here, the 3-beam case has the same triangular lattice as the 6-beam case, while the 4-beam case has a square lattice. As it is obvious that the lattice arrangement of the chiral structure influences its properties as a metasurface, we are interested in chiral structure formation when a 4-beam, i.e. a square lattice, is used.

In this study, the macroscopic optical radiation force equation is derived from the Lorentz force equation, and the optical radiation force distributions for a Gaussian beam, with which chiral structures have been fabricated successfully, are simulated. Through a comparison with the geometries of square- and triangle-lattice optical spot arrays that correspond to 4- and 6-beam interference patterns, the required conditions for formation of chiral structures in an array are discussed.

## Theory

**Figure 1:**
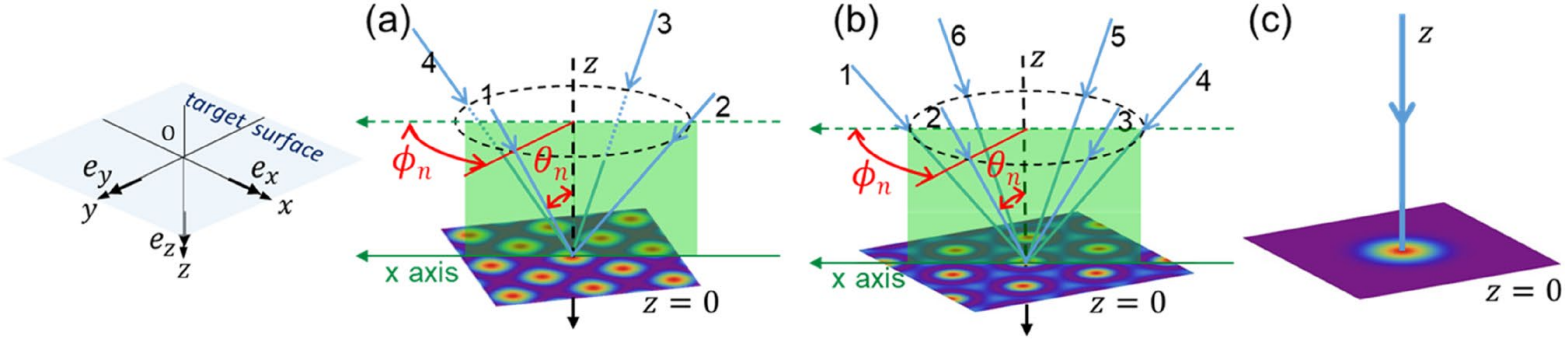
Scheme of light irradiation for (**a**) 4-beam interference, (**b**) 6-beam interference, and (**c**) a Gaussian beam. The left sketch shows the Cartesian system and unit vectors.

The scheme of the light irradiation using a 4-beam interference pattern, 6-beam interference pattern, and Gaussian beam is explained in Fig. 1a–c. In the simulation of the optical radiation force distribution with a 6-beam interference pattern, the similarity between the spot shape of the spot in an interference pattern and the spot shape for the Gaussian beam was used in a previous paper^16^. However, this method cannot be used for the 4-beam interference pattern because the spot shape is completely different from that of the Gaussian beam, as described below. In this paper, we calculate the optical radiation force distribution using a general description that is not limited to Gaussian beams^20^. Here, the calculations were performed in the Cartesian coordinate system, as shown in the left sketch in Fig. 1.

The macroscopic optical radiation force ${\varvec{F}}$ induced by an electromagnetic field, such as that generated by laser irradiation on a dielectric material, is expressed as follows:

1 $$\varvec{F} = \langle \rho _{p} \user2{E} + \user2{j}_{p} \times \user2{B}\rangle ,$$

where ${\varvec{E}}(x,y,z,t)$ is the complex electric field vector, ${\varvec{B}}$ is the complex magnetic flux density vector, ${\rho }_{p}$ is the polarized charge, ${{\varvec{j}}}_{p}$ is the polarization current, and $\langle \rangle$ indicates the time average. Consider the case of an isotropic and homogeneous medium (${\rho }_{p}=0$). The relationship of the complex susceptibility with other relevant variables is expressed by the following equation:

2 $${{\varvec{j}}}_{p}=\chi {\varepsilon }_{0}\frac{\partial {\varvec{E}}}{\partial t}=-i\omega \chi {\varepsilon }_{0}\varvec{E},$$

where $\chi \left(={\chi }_{r}+i{\chi }_{i}\right)$ is the complex susceptibility, ${\varepsilon }_{0}$ is the dielectric constant in vacuum, and $\omega$ is the angular frequency of the electric field. From these two equations, we can obtain

3 $$\begin{aligned}{\varvec{F}}&=\langle -i\omega \chi {\varepsilon }_{0}{\varvec{E}}\times {\varvec{B}}\rangle \\& =\frac{\omega {\varepsilon }_{0}}{2}\left\{{\chi }_{r}\mathrm{Im}\left[{\varvec{E}}\times {{\varvec{B}}}^{\boldsymbol{*}}\right]+{\chi }_{i}\mathrm{Re}\left[{\varvec{E}}\times {{\varvec{B}}}^{\boldsymbol{*}}\right]\right\}. \end{aligned}$$

The magnetic flux density vector ${\varvec{B}}$ is expressed according to the following equation (derived from Maxwell's equation):

4 $$\varvec{B}=-\frac{i}{\omega }\nabla \times \varvec{E}.$$

Then, Eq. (3) is.

5 $$\varvec{F}=-\frac{{\varepsilon }_{0}}{2}\left\{{\chi }_{r}\mathrm{Re}\left[\nabla \left({\varvec{E}}\cdot {{\varvec{E}}}^{*}\right)-\left({\varvec{E}}\cdot \nabla \right){{\varvec{E}}}^{*}\right]+{\chi }_{i}\mathrm{Im}\left[\nabla \left({\varvec{E}}\cdot {{\varvec{E}}}^{*}\right)-\left({\varvec{E}}\cdot \nabla \right){{\varvec{E}}}^{*}\right]\right\}$$

Here, using the Jones vector ${\varvec{J}}\left(=({J}_{x}, {J}_{y}), {\left|{J}_{x}\right|}^{2}+{\left|{J}_{y}\right|}^{2}=1\right)$,

6 $$\nabla \left({\varvec{E}}\cdot {{\varvec{E}}}^{*}\right)-\left({\varvec{E}}\cdot \nabla \right){{\varvec{E}}}^{*} =\frac{1}{2}\left({\varvec{J}}\cdot {{\varvec{J}}}^{*}-{{\varvec{J}}}^{*}{{\varvec{J}}}^{T}\right)\nabla {\left|{\varvec{E}}\right|}^{2} =\frac{1}{2}\left({\varvec{I}}-{{\varvec{J}}}^{*}{{\varvec{J}}}^{T}\right)\nabla {\left|{\varvec{E}}\right|}^{2},$$

where ${\varvec{I}}$ is the identity matrix, and ${{\varvec{J}}}^{T}$ is the transpose of ${\varvec{J}}$. Let $s=1, -1$ express right- or left-handed circular polarization, respectively, and the Jones vector is ${\varvec{J}}=1/\sqrt{2}(1, is)^{T}$. Using this and Eqs. (5) and (6), the optical radiation force in the case of circularly polarized light is expressed by the following equation:

7 $$\varvec{F}=\frac{{\varepsilon }_{0}}{8}\left[\left({\chi }_{r}\frac{\partial {\left|{\varvec{E}}\right|}^{2}}{\partial x}-s{\chi }_{i}\frac{\partial {\left|{\varvec{E}}\right|}^{2}}{\partial y}\right){{\varvec{e}}}_{x}+\left({\chi }_{r}\frac{\partial {\left|{\varvec{E}}\right|}^{2}}{\partial y}+s{\chi }_{i}\frac{\partial {\left|{\varvec{E}}\right|}^{2}}{\partial x}\right){{\varvec{e}}}_{y}\right],$$

where ${{\varvec{e}}}_{x}$ and ${{\varvec{e}}}_{y}$ are the unit vectors that represent the polarization. On the other hand, the Jones vector for linear polarization is ${\varvec{J}}=\left(1, 0\right)$, and the optical radiation force for linearly polarized light in the $x$-axis direction is expressed as follows:

8 $$\varvec{F}=\frac{{\varepsilon }_{0}}{4}{\chi }_{r}\frac{\partial {\left|{\varvec{E}}\right|}^{2}}{\partial y}{{\varvec{e}}}_{y},$$

where $I\propto {\left|{\varvec{E}}\right|}^{2}$ is the light intensity. Equations (7) and (8) allow us to simulate the optical radiation force vector for any light intensity distribution by calculating it. The method used to calculate the light intensity distribution for a given interference pattern is described in previous papers^21,22^.

**Figure 2:**
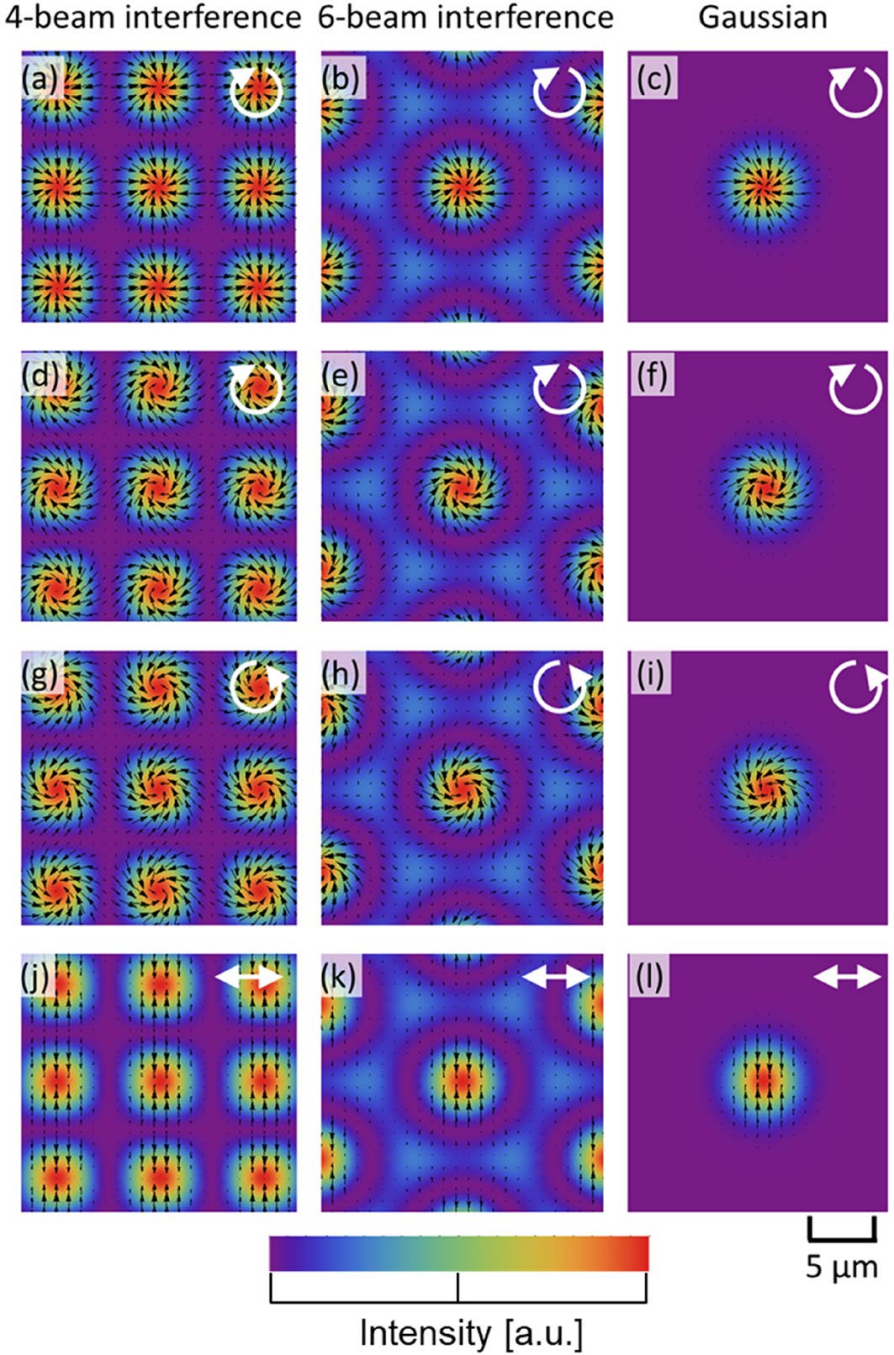
Optical radiation force distributions for a 4-beam, 6-beam, and Gaussian beam. The polarization is indicated on the images: (**a**–**f**) right-handed circular polarized, (**g**–**i**) left-handed circular polarized and (**j**–**l**) linearly polarized. In the simulation, $5{\chi }_{i}$ (instead of ${\chi }_{i}$) is applied in (**d**–**i**) to enhance the visibility of the azimuthal torque.

**Figure 3:**
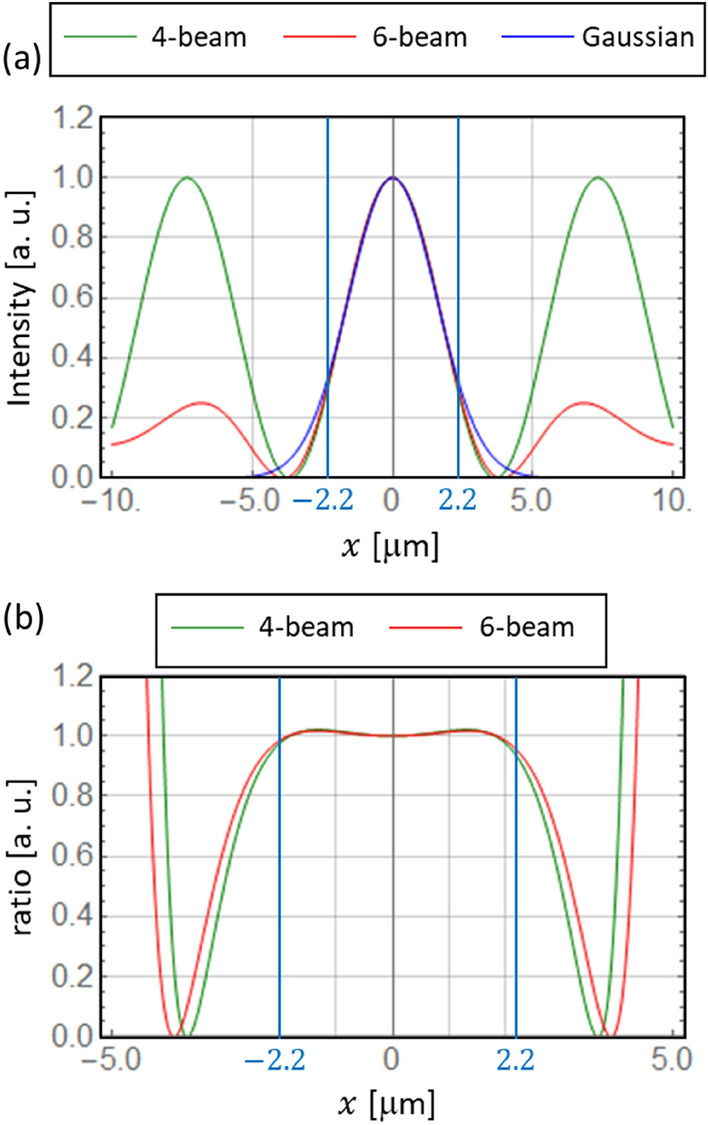
(**a**) Light intensity in the $x$-axis direction through the spot centre for the 4-beam and 6-beam interference patterns and the Gaussian beam. (**b**) Intensity ratio of the interference patterns to the Gaussian beam.

The optical radiation force distributions for a 4-beam interference pattern, a 6-beam interference pattern, and a Gaussian beam are shown in Fig. 2. The direction of polarization is indicated by an arrow on each figure. The black arrow represents the optical radiation force vector. The incident angle ${\theta }_{n}$, shown in Table 1, was used to ensure that the spot size in the interference pattern matched, within ± 5%, the radius of the Gaussian beam, as shown in Fig. 3a,b. ${\chi }_{r}$ and ${\chi }_{i}$ are the values of the polymer used in the experiment described below. In the simulation, except in Fig. 2a–c, ${5\chi }_{i}$ is used to emphasize the direction of the rotation of the optical radiation force vector.

**Table 1:** Parameters used in the simulations of the optical radiation force and light intensity distribution shown in Fig. 2.

Parameters	${E}_{0}$ (a. u.)	$\lambda$ (nm)	${\theta }_{n}$ (deg.)	$\Delta \phi$ (deg.)	$s$	${\chi }_{r}$	${\chi }_{i}$
4 beams	1.0	488	2.70	90.0	$0, \pm 1$	1.156	0.202
6 beams	1.0	488	2.73	60.0	$0, \pm 1$	1.156	0.202

Parameters	${E}_{0}$ (a. u.)	$\lambda$ (nm)	${w}_{0}$ (µm)	–	$s$	${\chi }_{r}$	${\chi }_{i}$
Gaussian	1.0	488	2.20	–	$0, \pm 1$	1.156	0.202

In the case of circularly polarized light, it can be seen that for both interference patterns and Gaussian beams, the optical radiation force is distributed such that it gathers at the centre of the spot while rotating in the same direction as the polarization. This is the cause of the formation of the chiral structure. In the experiments shown in a previous paper using the Gaussian beam and 6-beam interference pattern, the chiral structure was formed by this optical radiation force distribution^5,16^. In addition, the optical radiation force is zero at the spot centre and between the spots. This is one of the reasons that chiral structures have not formed in the centre of a spot in past experiments, as discussed later.

On the other hand, in the case of linearly polarized light, the optical radiation force is distributed to press in the direction perpendicular to the polarization, which is also in accordance with the previous experiment^16^. From these results, it appears that the formation of a chiral structure can be expected for the 4-beam interference pattern as well as for the Gaussian beam, but this is not the case in this experiment, as shown in the next section. To investigate the mechanism for this result, a more detailed analysis is given below.

**Figure 4:**
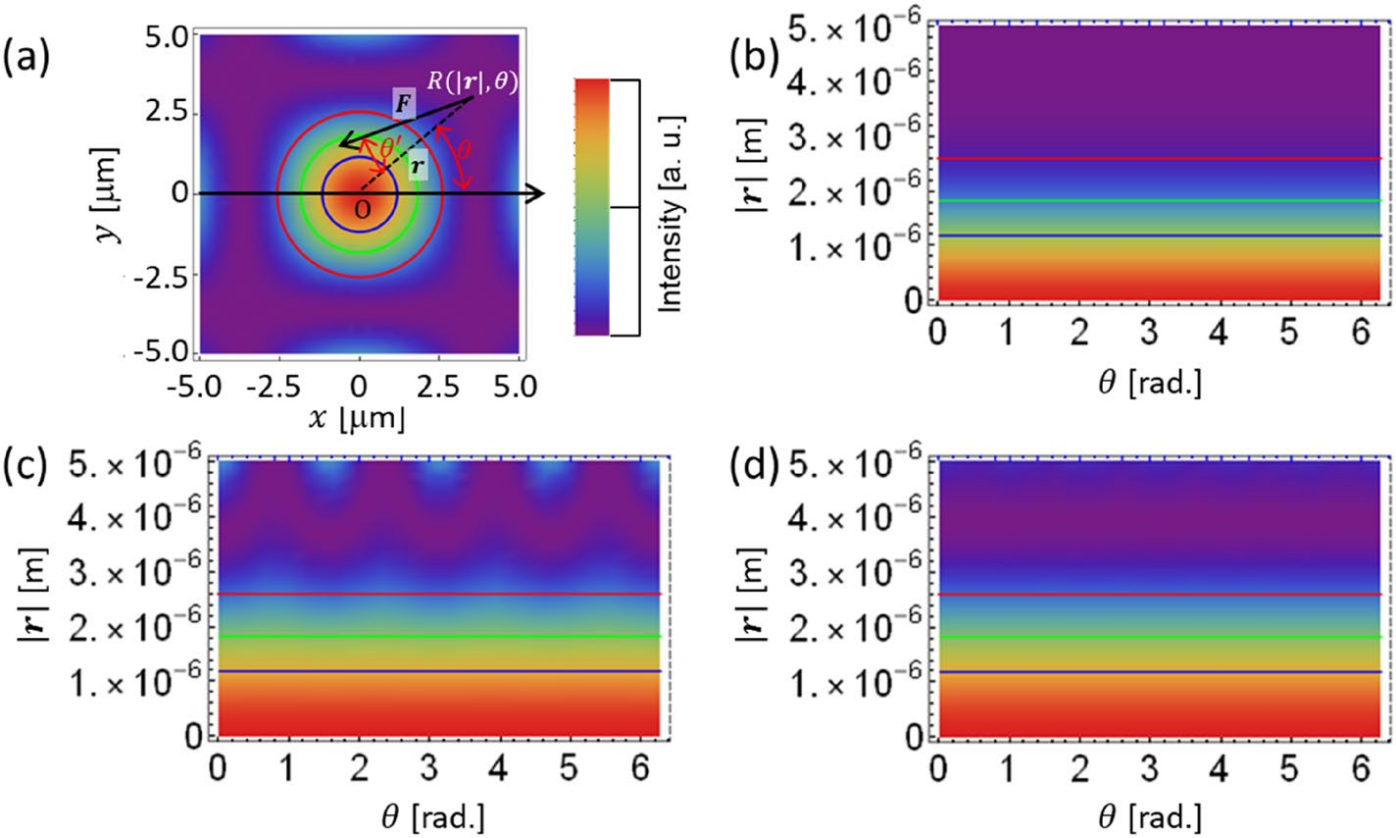
(**a**) Explanation of the light intensity distribution in a spot. This example corresponds to a 4-beam interference pattern. The concentric blue, green, and red circles represent $\left|{\varvec{r}}\right|=1.18, 1.83,$ and $2.59$ μm, respectively. An explanation of the deflection angle $\theta \mathrm{^{\prime}}$ of the radiation force ${\varvec{F}}$ at $R(r,\theta )$ is shown. (**b**–**d**) Variation of the light intensity in a Gaussian spot and 4- and 6-beam interference patterns versus declination angle $\theta$ and $\left|{\varvec{r}}\right|$, respectively.

**Figure 5:**
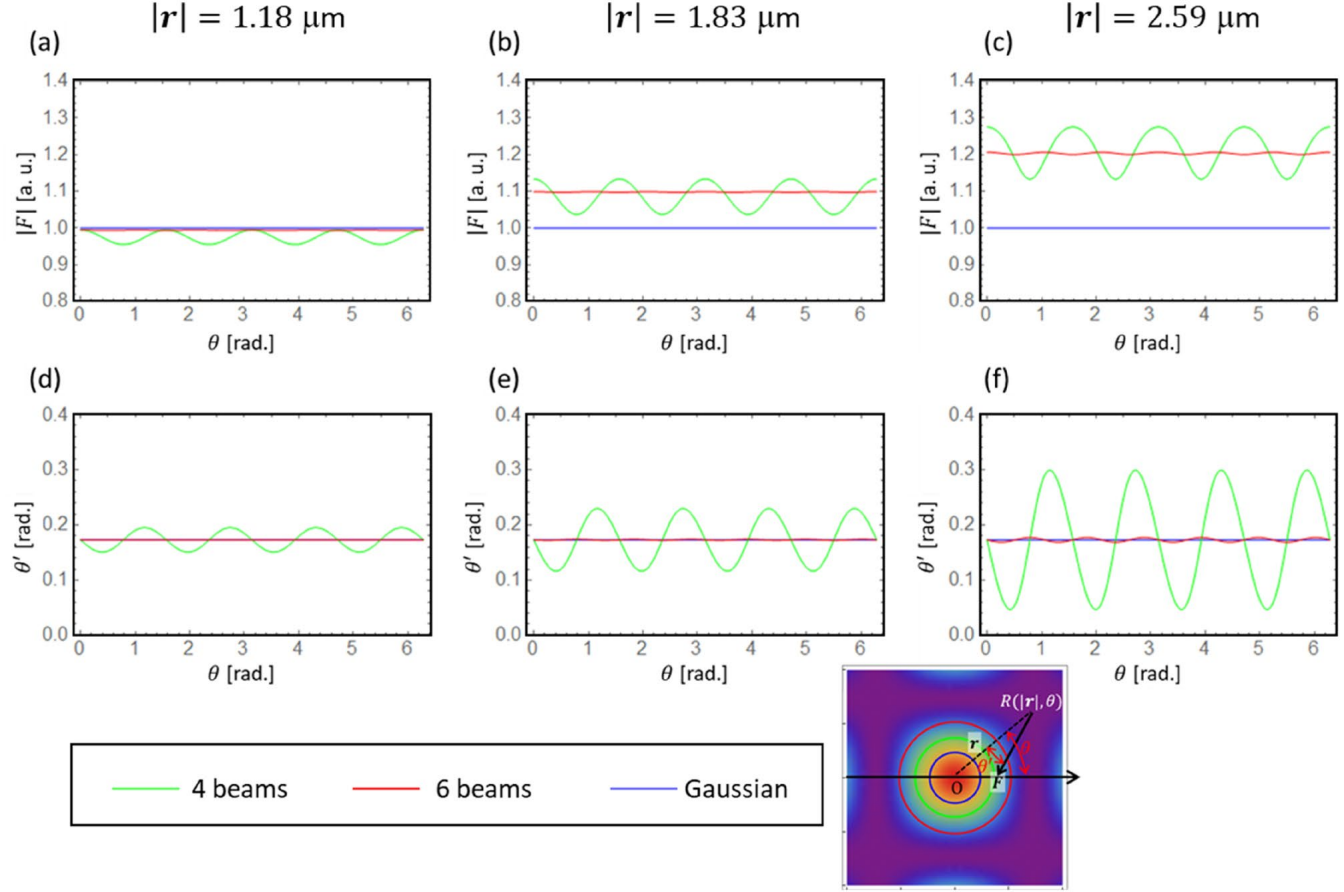
(**a**–**c**) Optical radiation force $\left|{\varvec{F}}\right|$ and (**d**–**f**) deflection angle $\theta \mathrm{^{\prime}}$ for different light distribution conditions: 4-beams, 6-beams and Gaussian beam. The optical radiation force is standardized to that of the Gaussian beam.

Figure 4a explains the light intensity distribution of a spot. ${\varvec{F}}$ is an optical radiation vector at $R(\left|{\varvec{r}}\right|,\theta )$ in a cylindrical coordinate system. Here, $\theta \mathrm{^{\prime}}$ denotes the deflection angle from the $R\mathrm{O}$ vector. Figures 4b–d show the variation of the light intensity in a Gaussian spot and 4- and 6-beam interference patterns versus the declination angle $\theta$ and $\left|{\varvec{r}}\right|$, respectively. It seems that there is no declination dependence in the light intensity distribution for a Gaussian beam or a 6-beam interference pattern. In contrast, a 4-period 'swell' is present as shown in Fig. 4c, which is consistent with the slightly rectangular spot shape shown in Fig. 4a. The blue, green, and red lines shown in Fig. 4 indicate $\left|{\varvec{r}}\right|=1.18, 1.83,$ and $2.59$ μm, where the light intensity of the Gaussian beam is 75%, 50%, and 25% of the peak value. Next, $\left|{\varvec{F}}\right|$ and $\theta \mathrm{^{\prime}}$ on these colour lines on the declination angle $\theta$ is plotted in Fig. 5.

Figure 5a–c shows the results of numerical calculations and the dependence of the optical radiation force $\left|{\varvec{F}}\right|$ on the declination angle $\theta$. Firstly, the optical radiation force has no dependence on $\theta$, at any $\left|{\varvec{r}}\right|$, for the Gaussian beam. However, for $\left|{\varvec{r}}\right|$ = 1.18 μm, which is relatively close to the centre (Fig. 5a), the optical radiation force of the 4-beam interference pattern shows that there are four periods of undulation during one period of $\theta$. The amplitude of the swell increases, as shown in Fig. 5b,c, meaning that the further away a given position is from the centre, the larger the dependence of the optical radiation force on $\theta$ becomes. In the case of the 6-beam interference pattern, almost no undulation can be seen, as in the case of the Gaussian beam. The $\theta$ dependence of the deflection angle ${\theta }^{^{\prime}}$ of the optical radiation force is plotted in Fig. 5d–f. 4-period undulations exist in the 4-beam interference pattern, as in the case of the optical radiation force, and the undulation increases as it becomes more distant from the centre. The deflection angle in the case of the Gaussian beam and the 6-beam interference pattern does not change with $\theta$, and the spiral inward optical radiation force distribution is maintained in a clockwise fashion.

In summary, the optical radiation force and its deflection angle are constant with respect to $\theta$ for the Gaussian beam and 6-beam interference pattern, while they are non-uniform for the 4-beam interference pattern due to the presence of undulation. Considering past successes in chiral structure formation using Gaussian beams^5,23,24^, it can be hypothesised; given a vector ${\varvec{r}}$ with the centre of the light spot at $\mathrm{O}$ and polar coordinates $R(\left|{\varvec{r}}\right|, \theta )$, and an optical radiation force vector ${\varvec{F}}$ at $R$, the deflection angle $\theta \mathrm{^{\prime}}=\mathrm{\angle }({\varvec{r}},{\varvec{F}})$ and $\left|{\varvec{F}}\right|$ must be constant with respect to $\theta$ for the chiral structure to form. Next, we investigate the difference based on the experimental results with interference patterns. As Gaussian and 6-beam interference processing have been experimented^5,16^, 4-beam interference was firstly experimented.

## Results and discussion

**Figure 6:**
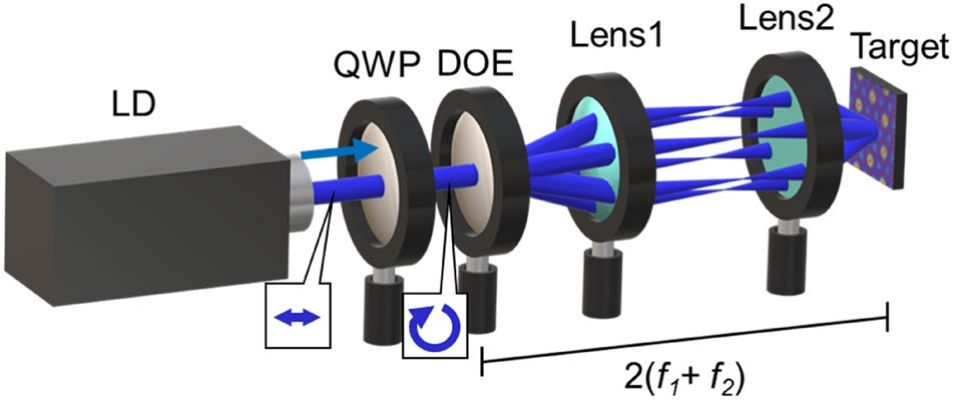
Experimental setup. *LD* CW laser diode, *QWP* quarter-wave plate, *DOE* diffractive optical element that diffracts 4- or 6-beams equally.

The experimental setup is shown in Fig. 6. A single-mode CW (continuous wave) laser was used, and the wavelength was 488 nm. The circularly polarized beam was split by a diffracted optical element (DOE) into 4 or 6 first-order diffracted beams. They are correlated on a surface of the target via a demagnification system that consists of two achromatic convex lenses. An azopolymer pDR1M thin-film target was used. The DOE and the target are in an image-transfer relationship, and both planes have the same polarization.

**Figure 7:**
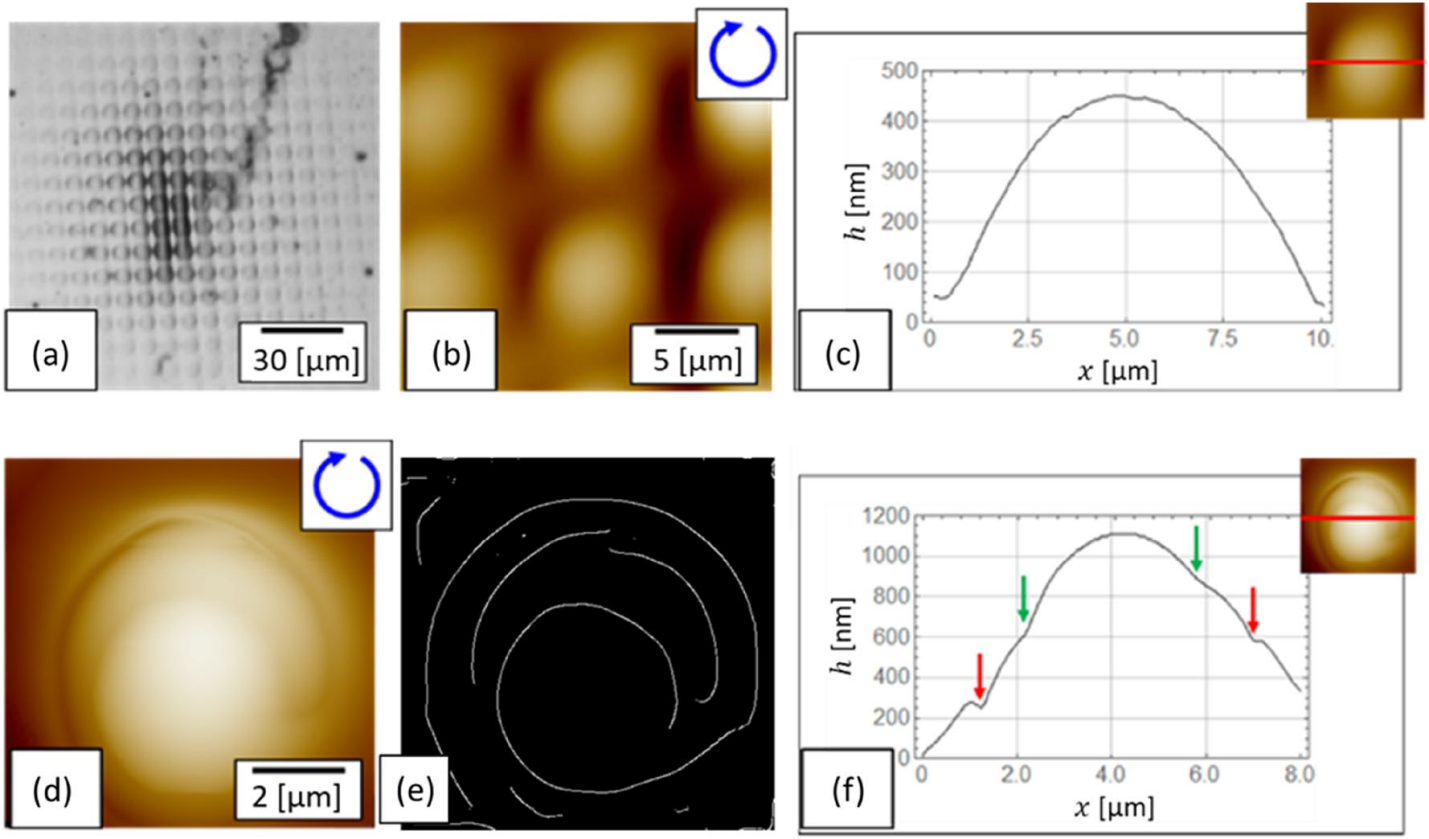
Surface morphology of the structure fabricated by (**a**–**c**) 4-beam and (**d**–**f**) 6-beam interference patterns. A right-handed circular polarized beam was used in both cases. (**a**) Optical image, (**b**,**d**) AFM images, (**c**,**f**) cross-sectional graphs of (**b**) and (**d**), (**e**) edge detection processed image of (**d**). (**d**,**f**) are reproduced from reference 16. Copyright 2018, Nature Publishing Group.

Figure 7a–c shows the structure fabricated on the target using an interference pattern of circularly polarized 4-beams. The average power density was 76.2 W/cm^2^ and the exposure duration was 5 s; these parameters were chosen to prevent the periodic structure from being melted and destroyed. As shown in an optical image (Fig. 7a), a regular square periodic structure with $\Lambda =8.4$ μm was fabricated; this was in accordance with the interference pattern. In the AFM (atomic force microscope) image (Fig. 7b), convex structures arranged in regular square lattices can be observed. Figure 7c shows a cross-sectional plot along the red line across the centre of a spot, and no steps due to the helical structure were observed. This result is completely different from that obtained with the 6-beam interference pattern in the previous experiment^16^. In Fig. 7d,f, a spiral structure was formed, and clear steps can be seen in Fig. 7f, as indicated by the red and green arrows. Figure 7e is an image of the detected edges in Fig. 7d, which clearly shows the spiral structure.

The conditions that must be met for a chiral structure to be formed are discussed based on the comparison of the experimental and simulation results. In the simulations, $\theta \mathrm{^{\prime}}=\mathrm{\angle }({\varvec{r}},{\varvec{F}})$ and $\left|{\varvec{F}}\right|$ must be constant with respect to $\theta$ to fabricate chiral structure. It is fulfilled in the case of 6-beam interference pattern, while they are non-uniform for the 4-beam interference pattern, which are consistent with the experimental results. In summary, these requirements are assumed to be necessary for the creation of chiral structures with interference patterns.

This phenomenon can be intuitively understood by considering it as analogous to stirring a viscous fluid with a magnetic stirrer. A coin-type stirrer, which applies uniform torque at $\left|{\varvec{r}}\right|$ at all $\theta$ to the fluid, results in a very uniform rotation of the fluid. On the other hand, a rod-type stirrer, which exerts torque on the fluid at only a few points, causes the fluid to have a relatively non-uniform behaviour. A similar phenomenon may be occurring in azopolymer deformation: the non-uniform torque distribution may eliminate the chiral structure. In any case, the control of the spot shape in interference patterns is likely to be the key to forming an arrayed chiral structure.

Here, the transmission of optical radiation pressure at adjacent spots in the interference pattern may make the formation of chiral structure mechanism more complicated. In this case, the transmitting pressure is more uniform with respect to $\theta$ in the case of 6-beam interference than for 4-beam interference. Hence, this hypothesis is consistent with the experimental difficulties in relation to forming chiral structures in 4-beam interference patterns.

One point to note is that there is no chiral structure in the middle of the spot, even though a uniform $\left|{\varvec{F}}\right|$ and $\theta \mathrm{^{\prime}}$ are obtained under all conditions. This is true not only for interference patterns but also for experiments using a Gaussian beam. Possible reasons for this include the loss of fine grooves in the chiral structure of the polymer due to the high irradiation intensity and temperature at the centre, and the low light intensity gradient at the centre, which results in low light radiation pressure, as shown in Fig. 2.

## Conclusion

In this study, the conditions for the formation of chiral structures using interference patterns, as well as the formation of chiral structures with Gaussian beams, are investigated using simulations and experiments. It seems that the magnitude and declination of the optical radiation force need to be constant with respect to *θ* to form a chiral structure, as in the case of Gaussian beams. This condition is fulfilled with an interference pattern with 6-beams rather than 4-beams, and this hypothesis was supported by the experimental results. It should be noted that a vast number of parameters, such as the dielectric constant, complex susceptibility, target and substrate structures, and laser irradiation conditions have not been investigated, meaning that the possibility of forming square-lattice-array chiral structures using 4-beam interference patterns cannot be completely ruled out.

The simulation of the optical radiation force distribution uses a method that allows the simulation of any light intensity distribution. This can be combined with techniques not only for controlling Gaussian beams but also for designing spot shapes and interference patterns. Furthermore, simulations that take the spatio-temporal variation of these patterns into account are possible.

Within the range of the required conditions’, it is possible to enhance the properties and phenomena of the chiral structure by arraying, and this study will contribute to the development of chiral devices, which are in the early stages of basic research.

## Methods

### Simulation^25^

In the simulation of the optical radiation force distributions, Eqs. (7) and (8) are expressed as functions in Wolfram Mathematica. The expressions for the light intensity distribution of the interference patterns are shown in previous papers^21,22^.

### Preparation of target^26^

pDR1M (Poly(Disperse Red 1 methacrylate)), Sigma-Aldrich) acetone solution (10%) is suspended on a plasma-cleaned (3 min) silica glass substrate. A drop of toluene is added to this solution to enhance spreadability; then, it is dried in atmosphere at room temperature.

### Experimental setup

A single-mode CW laser beam from a Sapphire 488 SF (Coherent Inc.) operated at 488 nm passed through a $\lambda /4$ waveplate to transform the polarization to right-handed circular polarization. A DOE diffracted 4-beams with the same intensity and correlated them on a target via two achromatic lenses. The experiment was performed in air at room temperature. The resultant structure was measured using an AFM (VN-8000, KEYENCE) and imaged by an optical microscope attached to the AFM. The edge detection process shown in Fig. 7e was performed using Mathematica's EdgeDetect function.

## Data Availability

The datasets generated during and/or analysed during the current study are available from the corresponding author on reasonable request.
